# Sugar and Teeth

**Published:** 1882-10

**Authors:** 


### SUGAR AND TEETH.

In a previous number it was stated that pure sugar and candies, having no residue, could not, by lodgement about the teeth, injure them; and that if used in moderation, neither sugar nor candies were prejudicial to the teeth or health of young children or grown persons; that there was more or less sugar in all vegetable food; but as concentrations were liable to abuse, we advised that they should be taken at regular meals.

The *Medical Journal* of Charleston, S. C., states the conclusions of M. Larez:

1st. Refined Sugar injures teeth, either by immediate contact, or by gas developed in the stomach.

2d. That a tooth soaked in sugar water, becomes jelly like, from the sugar combining with the lime of the tooth.

